# Genetic structure as a response to anthropogenic and extreme weather disturbances of a coastal dune dwelling spider, *Arctosa sanctaerosae*

**DOI:** 10.1002/ece3.6919

**Published:** 2020-12-31

**Authors:** Robert A. Hataway, David H. Reed

**Affiliations:** ^1^ Samford University Birmingham AL USA

**Keywords:** coastal conservation, dune habitat, effective population size, hurricane

## Abstract

The continued increase in the number of tourists visiting the Northern Gulf Coast (NGC), USA, in the last century, and the resulting sprawl of large cities along the coast, has degraded and fragmented the available habitat of *Arctosa sanctaerosae,* a wolf spider endemic to the secondary dunes of the white sandy beaches of the NGC. In addition to anthropogenic disturbance to this coastal region, hurricanes are an additional and natural perturbation to the ecosystem. The data presented here explore the status of populations of this species spanning the entire known range and the factors influencing population demography including anthropogenic disturbance and severe tropical storms. Using microsatellite markers, we were able to document the genetic structure of *A. sanctaerosae*, including current and historic patterns of migration. These results combined with ecological and census data reveal the characteristics that have influenced population persistence: ecological variables affecting the recovery of the population clusters after severe tropical storms, genetic fragmentation due to anthropogenic disturbance, and their interaction. These findings demonstrate the significance that the high traffic beach communities of the NGC and their impact on the once intact contiguous dune ecosystem have on recovery after severe tropical storms. Contemporary modeling methods that compare current and historic levels of gene flow suggest *A. sanctaerosae* has experienced a single, contiguous population subdivision, and the isolates reduced in size since the onset of commercial development of the NGC. These results point to the need for monitoring of the species and increased protection for this endangered habitat.

## Graphical abstract

The data presented here explore the status of populations of a *Arctosa sanctaerosae*, a dune dwelling spider, spanning the entire known range and the factors influencing population demography including anthropogenic disturbance and severe tropical storms. Using microsatellite markers, we were able to document the genetic structure of *A. sanctaerosae*, including current and historical patterns of migration. These results combined with ecological and census data reveal the characteristics that have influenced population persistence: ecological variables affecting the recovery of the population clusters after severe tropical storms, genetic fragmentation due to anthropogenic disturbance, and the interaction between these.

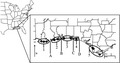

## 1 INTRODUCTION

Human degradation of habitat is known to interrupt migration between subpopulations through fragmentation and can influence population viability (Reed, 2004; Reed, Lowe, et al., 2003; Reed, O'Grady, Ballou, et al., 2003; Reed, O'Grady, Brook, et al., 2003). This human degradation represents a novel disturbance to species that evolved in the absence of anthropogenic factors (Pickett & White, 1985). The high rate at which human encroachment occurs may prevent the evolution of behaviors of life history traits to avoid extinction or extirpation (Boulding & Hay, 2001; Stockwell et al., 2003). In addition to human pressures, naturally occurring extreme environmental perturbations (catastrophes) have a profound effect on the persistence time of populations and species (Brooks & Smith, 2001; Lande, 1993; Mangel & Tier, 1993; Reed, 2007; Reed, Lowe, et al., 2003; Reed, O'Grady, Ballou, et al., 2003; Reed, O'Grady, Brook, et al., 2003; Schoener et al., 2001; Spiller et al., 1998; Young, 1994). There have been few studies that look at the role of hurricanes as a form of disturbance regime and their effects on population dynamics (Askins & Ewert, 1991; Lande, 1993; Lynch, 1991; Reagan, 1991; Reed, Lowe, et al., 2003; Reed, O'Grady, Ballou, et al., 2003; Reed, O'Grady, Brook, et al., 2003; Traylor‐Holzer et al., 2005; Waide, 1991; Willig & Camilo, 1991; Woolbright, 1991; Wunderle et al., 1992).

The interaction of the hurricanes and human‐induced pressures on coastal taxa has not been extensively studied. Human modifications are known to severely limit the dynamic ability of an ecosystem for vary naturally (Nordstrom, 2000) or can amplify the impacts of naturally occurring stochastic disturbances (Jonzen et al., 2004; Schrott et al., 2005). These modifications include alteration to the supply and transport of the sand as well as climate change‐induced sea level and surface temperature rise which is predicted to increase the severity of tropical storms (Komar, 1998; Slott et al., 2006).

The ecosystem along the Northern Gulf of Mexico Coast (NGC) is experiencing growth in both of these classes of disturbance. The NGC including Northern Florida, Alabama, Mississippi, and Louisiana has had 35 hurricanes of category 3 or higher make landfall in the last 100 years and in that same period, the human population of the major cities along NGC has increased approximately 15‐fold from 10,000 individuals to 150,000 (U. S. Census Bureau, 1990). This increased habitat fragmentation due to human encroachment is hypothesized to have reduced population size of flora and fauna through the creation of barriers to migration and subsequent subdivision of larger populations into a series of smaller populations. Small populations experience reduced population viability and persistence (Palstra & Ruzzante, 2008; Reed, 2010; Reed, Lowe, et al., 2003; Reed, O'Grady, Ballou, et al., 2003; Reed, O'Grady, Brook, et al., 2003; Saccheri et al., 1998). Small populations are also more susceptible to loss of genetic diversity due to random genetic drift, they maintain lower levels of fitness (Reed, 2005; Reed & Frankham, 2003), and have reduced adaptive potential (Blows & Hoffman, 2005; Reed, 2005; Reed, Lowe, et al., 2003; Reed, O'Grady, Ballou, et al., 2003; Reed, O'Grady, Brook, et al., 2003) when compared to larger populations.

The spatial heterogeneity in hurricane impacts suggests that spatial autocorrelations in population fluctuations (Burgman et al., 1993; McCarthy & Lindenmayer, 2000; Reed, 2004) might be especially important to metapopulation persistence in this system. Because hurricanes reduce population size via the direct destruction of habitat, results from one habitat‐specific species with similarities in vulnerability to storm‐driven mortality should be relevant to the persistence of all species limited to that habitat.

**Figure 1:**
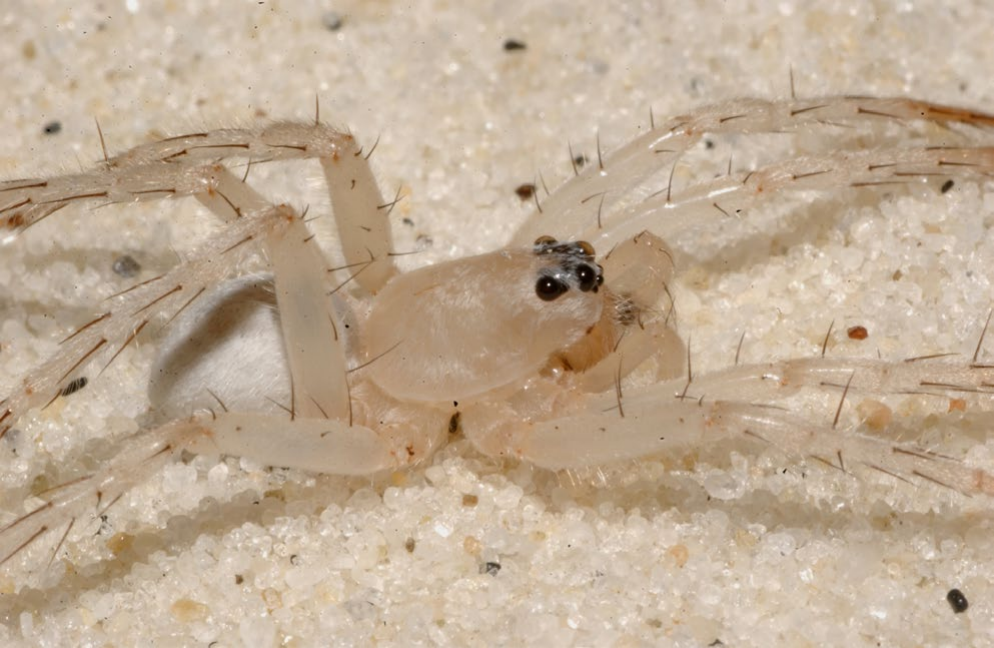
*Arctosa sanctaerosae,* Gertsch and Wallace, 1935 (Araneae: Lycosidae)

Using the nocturnal burrow‐dwelling wolf spider, *Arctosa sanctaerosae,* Gertsch and Wallace, 1935 (Araneae: Lycosidae) (Figure 1), we are able to investigate the effects of habitat fragmentation (human encroachment) and a catastrophe regime (severe tropical storms) as well as the interaction of these natural and anthropogenic disturbances. This species is an ideal subject to explore these questions, as it is entirely restricted to the secondary dunes in the coastal dune system of the NGC (McNatt et al., 2000). Given the general lack of invertebrate conservation work (Skerl & Gillespie, 1999) and the discrete generation length that spiders have, this taxon will provide insight into threats faced by other invertebrates and small vertebrate species of interest in the region (e.g., several species of the beach mouse *Peromyscus polionotus,* Osgood, 1907).

The purpose of this study was threefold: (a) explore the effects of severe tropical storms on spider density, and relate these to the physical attributes of the dune system, extent of disturbance, and the distance from the point of landfall of the tropical storm; (b) describe the genetic diversity and structure of these population clusters; and finally (c) to investigate gene flow, past and present, among these. The comparison of historic versus recent gene flow can potentially be used to differentiate isolation of populations caused by hurricanes and other forms of prehuman isolation (historically constant) versus contemporary causes of reduced connectivity including those caused by conversion of habitat by humans within the last 100 years. Assuming that hurricanes have occurred since the evolution of this species and that human disturbance on a large scale in the region started only a hundred years ago, the dominant force that is shaping the current status of the species and its populations should emerge. Gaining insight into the effects of environmental perturbations, anthropogenic encroachment, and their interaction would be invaluable informing the long‐term conservation goals species who share the same endangered coastal dune habitat along the NGC.

## 2 METHODS

### 2.1 Population densities and growth rates

**Figure 2:**
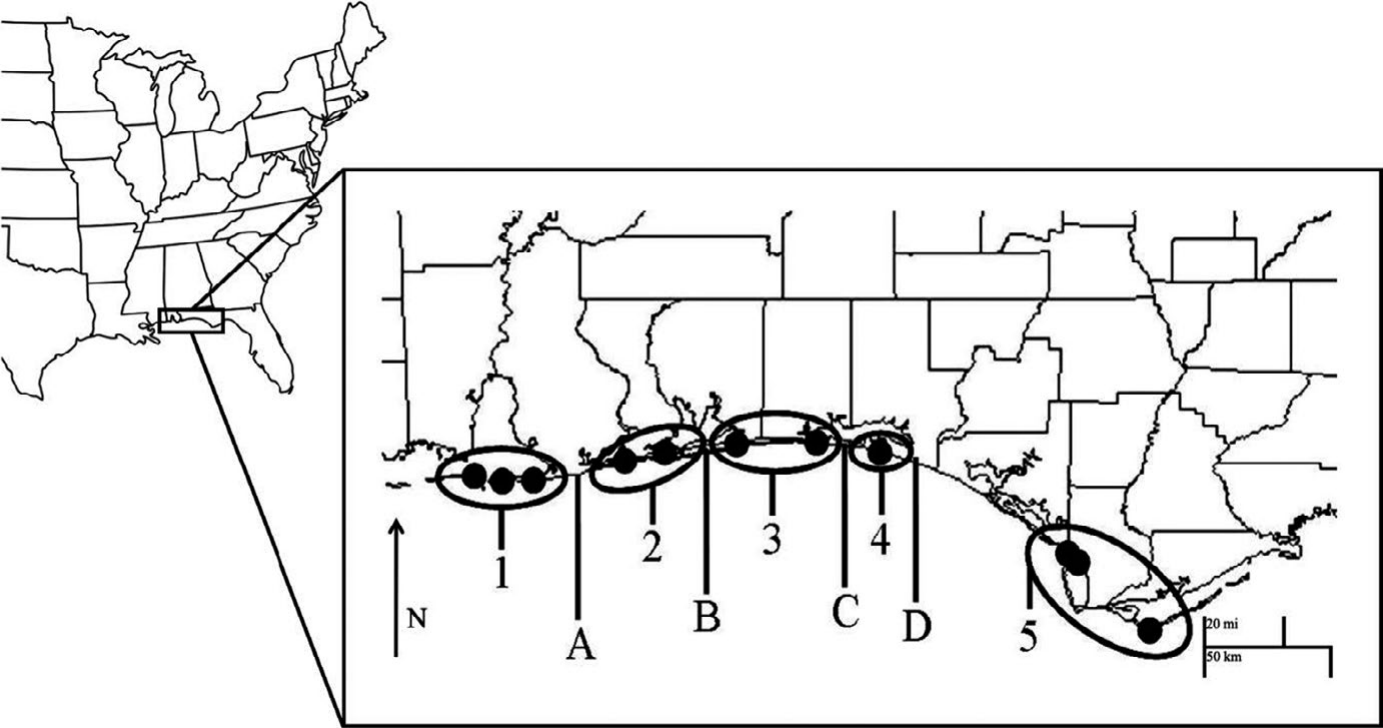
The five population clusters recovered from genetic data using GENELAND and STRUCTURE in circles with major human developments labeled: (a) Gulf Shores, AL, (b) Pensacola Beach, FL, (c and d) Destin, FL

Densities were measured by hand collecting individuals inside three independent 12 m by 12 m quadrats (144 m^2^) randomly placed within the secondary dunes of ten sites across the NGC during the summer months between 2003 and 2007 (Figure 2). Counts were made of spiders at or near their burrows one hour after nightfall on three consecutive clear nights. The quadrats were located each year using GPS data and resampled. Growth rate (*R*) was calculated as *R* = ln (*N*_1_/*N*_0_) for each site. All density estimates of zero were adjusted to 0.5 to ease statistical analysis based on the assumption that these population numbers were most likely not zero but too small to be detected.

These rates were compared before and after landfall of major hurricanes, Hurricane Ivan in 2004 and Hurricane Katrina in 2005. These hurricanes led to severe erosion of the dunes of the NGC. Hurricane Ivan made landfall at Gulf Shores, Alabama, on 16 September 2004 as a category three hurricane. Then, on 25 August 2005, Hurricane Katrina made its original landfall in southeast Louisiana as a category three hurricane. Densities were measured one month after landfall of the storms.

Physical measures, dune height (dh) and the density of vegetation, were also quantified within the quadrats. These two measures were included due to their provision and maintenance of habitat for *A. sanctaerosae* as well as prey. Vegetation cover of dunes was quantified by counting the number of stems of sea oats*, Uniola paniculata* L. (Liliopsida:Poaceae) and similar vegetation within three independent, randomly chosen one square meter quadrats within the larger quadrats on each of the three sampling nights per site. Dune height was measured from the height of the apparent high tide mark on the active beach to the highest point on the secondary due. Model selection for explaining population growth rates was accomplished using an information‐theoretic approach. Stepwise multiple regression was subsequently used to explore the relationships among these ecological factors and changes in them, as a function of hurricane landfall, associated with each site in order to identify those that best explained the variation in population decline and recovery observed after each of these two hurricanes.

### 2.2 Census population size

Census population sizes were calculated for each of the five clusters (recovered by genetic clustering algorithms detailed below) by multiplying population densities across the clusters by the total habitat area. The total extent of habitat was estimated by calculating the area of secondary dunes within each cluster. This was done using GOOGLE EARTH by creating polygons and using the area tool to calculate the total area. The density per quadrat was then multiplied against total area to estimate census population size.

### 2.3 Genetic sampling

Tissue samples from 20 individuals collected from each of the sites were collected between 1st June and 11th June 2007 to be used in genetic analyses. All individuals were stored at −80°C in 100% ethanol, and approximately 1 mg of tissue acquired from the legs was used for subsequent DNA extraction (Qiagen DNeasy kit). Target microsatellite sequences were amplified using 11 microsatellite primers, ten developed for the study species (Hataway et al., 2011) and an addition developed for a sister taxa. Fluorescence‐labeled fragments were visualized on an ABI 3130, and allele sizes were determined through comparison with a known size standard (GeneScan ‐500 ROX) using GENEMAPPER version 3.7. All scores were checked manually, and ambiguous fragments were reanalyzed.

### 2.4 Population structure

Population differentiation was accomplished using the Bayesian clustering algorithms in the programs STRUCTURE (Pritchard et al., 2000) and GENELAND ver. 3.2.4 (Guillot et al., 2005; Guillot & Santos, 2009). Both of these programs have idiosyncrasies and can be used in concert to not only suggest a current number of clusters and assign individuals to them, but also to find support for a suggested model. GENELAND was run and included the correlated model, which assigns individuals to geographic clusters without prior knowledge of the site where that individual was sampled. This accounts for several factors relevant to this system. Spatially, we expect areas of intense human impact to create a barrier between populations and so we add into the model the set of georeferenced coordinates, in this case the location of our collection sites (Guillot & Santos, 2009). GENELAND and STRUCTURE both recovered five clusters and these assignments were used for all subsequent demographic analyses.

### 2.5 Genetic diversity

FSTAT ver. 2.9.3.2 (Goudet, 2002) was used: (a) to test for Hardy–Weinberg equilibrium within populations (b) to estimate *F*_is_ (c) to calculate allelic richness (*A*_r_) and (d) to calculate gene diversity (*H*_s_). GENEPOP (Raymond & Rousset, 1995; Rousset, 2008) was used to test population differentiation and to estimate the number of null alleles. ARELEQUIN ver. 3.5.1.2 (Excoffier, 2010; Excoffier et al., 2005) was used to calculate expected (*H*_e_) and observed (*H*_o_) heterozygosities and pairwise *F*_st_ values between populations. Other statistics have been suggested for estimating gene flow in microsatellites (Goldstein & Pollock, 1997; Goldstein et al., 1995; Weir & Cockerham, 1984; Zhivotovsky, 1999), but *F*_st_ is the most commonly used metric and was employed here. Expected heterozygosity was computed among and within populations using Levene's method (1949) in the software package POPGEN (Yeh et al., 1999). Isolation by distance was tested using a Mantel test of log transformed linearized *F*_st_ and geographic distance values was conducted among the clusters.

### 2.6 Historic and recent geneflow estimation

Historic mutation scaled migration rates (M) were calculated using MIGRATE ver. 3.2.1 (Beerli, 2010). The estimates given are long‐term averages heavily influenced by the recent past and are calculated over the last 4*N*_e_ generations. The rate of recent gene flow was calculated using BAYESASS ver. 1.3 (Wilson & Rannala, 2003), which estimates migration rates within the last two to three generations using a Bayesian inference framework and gametic disequilibrium among immigrants and their descendants. Individuals from each of the five clusters were grouped suggested by GENELAND for both tests.

### 2.7 Historic and recent bottleneck detection

To test for evidence of bottlenecks, M ratios were calculated (Garza & Williamson, 2001) in ARLEQUIN version 3.1 (Excoffier, 2010; Excoffier et al., 2005). The effectiveness of this method has been shown to be maximized when the bottleneck is older and lasted several generations before recovery (Williamson‐Natesan, 2005).

BOTTLENECK (Cornuet and Luikart 1996; Luikart & Cornuet, 1998; Piry et al., 1999) was used for estimations of bottlenecks within the previous 4Ne generations, for this species approximately the last 100–500 years. The method employed in this program has been shown to have the ability to detect less severe and more recent reductions in population size.

### 2.8 Historic and recent effective population size estimation

Historic effective populations sizes were measured using three methods. First, using the equation Ө = 4Neμ where Ө is the mutation scaled effective population size and μ is the mutation rate (Gaggiotti & Excoffier, 2000). Mutation rate was held constant and at a rate of 5x10^‐4,^ and a coalescent approach was used to estimate of Ө over the last 4Ne generations in the program MIGRATE (Beerli, 2010; Beerli & Felsenstein, 1999).

Historic effective population size was also calculated using the methods of Hartl and Clark (1989) and Ohta and Kimura (1973). These methods assume an infinite allele model (IAM) and a stepwise mutational model (SMM), respectively. Both methods hold *N*_e_ as a function of *H*_e_. The mutational models estimate the upper and lower extremes of mutation, and so the true Ne is likely to be found between the two estimates (Busch et al., 2007). A paired *t* test was then carried out between the historic and recent estimates of Ne.

Recent effective population sizes were estimated using ONeSamp (Tallmon et al., 2008) as well as LDNe (Waples & Do, 2008). LDNe uses linkage disequilibrium to estimate *N*_e_. The major issue affecting its usefulness for this study is that linkage disequilibrium can be caused by inbreeding, substructure, or immigration. The first is known to be an issue in this species, and so the results of these tests bear careful scrutiny. ONeSAMP on the other hand uses eight different genetic parameters including: “the number of alleles divided by allele length range (Garza & Williamson, 2001), the difference of the natural logarithms of variance in allele length and heterozygosity (King et al., 2000), expected heterozygosity (Nei, 1987), number of alleles per locus, Wright's *F*_IS_ (Nei, 1987), the mean and variance of multilocus homozygosity, and the square of the correlation of alleles at different loci (Hill 1981)” (Tallmon et al., 2008). This is expected to provide more accurate results, although high inbreeding levels (*F*_IS_) may confound results. The results for both methodologies including estimates using both 0.01 and 0.02 for the lowest allele frequencies for the analysis in LDNe are reported.

## 3 RESULTS

### 3.1 Population densities and growth rates

The mean population growth rate (*R*) across sites during the period between 2003 and 2004, which included the effects of Hurricane Ivan, was −2.703 (*SE* = 0.517). In the following one‐year period (2004–2005), the growth rate across sites was 1.738 (*SE* of 0.416). This demonstrates a pattern of reduction and recovery of densities, assuming that the sites had been stable for some time before this. This assumption is based on the extended period of time prior to Hurricane Ivan (8 years) since the last major tropical storm made landfall and the protected status of the sites used. There was an increase in density at all sites in the period following Ivan's landfall between 2004 and 2005. However, population densities had not returned to the levels they were found at in 2003 prior to the landfall of Katrina.

The average distance of study sites from the landfall of Hurricane Ivan was 106 km, while for Hurricane Katrina it was 288 km. The furthest sites from Katrina were over 400 km away and experienced little or no population reduction and, in most cases, had positive population growth rates despite the landfall of the hurricane. When the density of *A. sanctaerosae* prior to Hurricane Ivan is regressed against the proportional reductions of density of *A. sanctaerosae* at each site (density before storm divided by density after storm), the results are found to be nonsignificant (*p* = .67, *R* ^2^ = .031). The same is true for Hurricane Katrina (*p* = .7127, *R* ^2^ = .018). This confirms what had long been assumed, the effects of major hurricanes are density‐independent which in this case means the density of spiders prior to landfall of a major hurricane has no apparent impact on the magnitude of the loss of density after the landfall of the storm.

Multiple regression models were created to explore what ecological factors varied with population reductions for each of the two storms independently. The factors included were as follows: distance from landfall, dune height and vegetation before landfall, and proportional loss of dune height and vegetation after landfall. The model that best described the effects seen from the storms only included the distance from the point of landfall in both the case of Hurricane Ivan (*R* ^2^ adjusted = .638, *p* = .002, AIC adjusted = 5.046, *w* = 0.880) (Table 1) and Katrina (*R* ^2^ adjusted = .0369, *p* = .014, AIC adjusted = 39.561, *w* = 0.922) (Table 2).

**Table 1:** Comparison of models describing the proposed factors contributing to proportional population reduction as a result of Hurricane Ivan

Model	AICc	*w*
Distance from Landfall of Eyewall	5.406	0.880
+ Dune Height before	9.425	0.118
+ Vegetation before	17.443	0.002
+ loss of Vegetation	32.005	1.474E^−06^
+ loss of Dune Height	59.770	1.379E^−12^

**Table 2:** Comparison of models describing the proposed factors contributing to proportional population reduction as a result of Hurricane Katrina

Model	AICc	*w*
Distance from Landfall of Katrina	39.5611	0.922
+Loss of Dune Height	44.5507	0.076
+Loss of veg	52.2326	0.002
+2005–2006 veg	63.7523	5.150E^−06^
+2005 Dh	93.7519	1.576E^−12^

Similar multiple regression models for the recovery of density included the possible explanatory variables: distance from landfall, dune height and vegetation minimums after storms, and proportional recovery of dune height and vegetation in the year following the storms landfall. The model that best predicted the recovery of population density of *A. sanctaerosa*e after Hurricane Ivan suggests that variation in the recovery of dune height in the year following the storm event best explains the variation in the recovery of the population (*R* ^2^ adjusted = .416, *p* = 012, AIC adjusted = 36.261, *w* = 0.887) (Table 3). In the recovery from Hurricane Katrina, the best model incorporated both the recovery of dune height and the distance from the place of landfall (*R* ^2^ adjusted = .595, *p* = .009, AIC adjusted = 22.214, *w* = 0.806) (Table 4).

**Table 3:** Comparison of models describing the proposed factors contributing to population growth rate in the year immediately following Hurricane Ivan

Model	AICc	*w*
Recovery of Dune Height	36.261	0.887
+ Distance from Landfall Ivan	40.649	0.099
+Dune Height minimum after storm	44.474	0.015
+Vegetation minimum after storm	58.645	1.222E^−05^
+Recovery of Vegetation	88.460	4.099E^−12^

**Table 4:** Comparison of models describing the proposed factors contributing to population growth rate in the year immediately following Hurricane Katrina

Model	AICc	*w*
Recovery of Dune Height	25.510	0.155
+ Distance from Landfall Katrina	22.214	0.806
+Vegetation minimum after storm	28.269	0.039
+Dune Height minimum after storm	41.979	4.115E^−05^
+ Recovery of vegetation	71.957	1.272E^−11^

The recovery of the sites after Hurricane Ivan did appear to display a level of density dependent growth. The proportional reduction explained 45% of the rate of growth in the year following the storm (*R* ^2^ = .452, *p* = .0332): The larger the proportion of reduction, the faster the rate of recovery. This result is logical considering the increased habitat availability at sites whose densities were impacted more severely leaving open habitat patches.

There was spectrum of effects on the density of *A. sanctaerosae* after the storms from zero in the most distal sites to severe at sites such as Fort Pickens, FL. The density of individuals at Fort Pickens experienced the most severe effects from Hurricane Ivan. Extensive flooding and overwashing of the western portion of Santa Rosa Island occurred where this collection site is located. It was sampled in 2005–2007 (it was not sampled in 2004. Access to the site was restricted) with zero individuals being located in the first 3 years after Ivan. It was not until 2007 that individuals were found at this site. This suggests either a local extinction or such a severe reduction that individuals were not detectable with our sample sizes. Other sites saw depressions in population density but not as severe or sustained as what was seen at Ft. Pickens, FL.

### 3.2 Census population size

Census population sizes were large and range from 71,000 to 315,000 individuals spread across the available habitat per cluster (Table 5). There was a significant difference between recent *N*_e_ and estimates of *N*_c_ for each of the clusters.

**Table 5:** Estimates of historic and recent effective population size (*N*_e_) and census population size (*N*_c_) for each of the five population clusters recovered from genetic data using GENELAND and STRUCTURE

Population cluster	Historic *N*_e_			Recent *N*_e_			Census Population Estimate
	Mean of three methods used (SMM, IAM, and MIGRATE)			ONeSAMP			
	Lower C.I.	Mean	Upper C.I.	Lower C.I.	Ne	Upper C.I.	
1	88.7	94.0	160.4	53.7	68.4	81.4	128,350.0
2	115.5	160.2	204.9	18.6	32.7	43.7	87,597.9
3	103.3	193.6	283.9	34.5	50.2	68.4	71,066.7
4	287.6	351.0	614.3	31.1	46.8	56.3	196,48.6
5	544.4	773.0	1,501.5	101.7	153.9	243.6	315,842.2

### 3.3 Population structure

GENELAND and STRUCTURE identified five population clusters that were consistent across all independent runs. The three sites of western Alabama were clustered as a single population (Cluster 1) despite Dauphin Island, AL and Fort Morgan, AL being separated by a 5 km stretch of open water. The sites combined to form both Clusters 2 and 3 are also separated by stretches of water. A single site, geographically isolated by human development composed Cluster 4. Finally, the three most easterly sites formed Cluster 5 (Figure 1).

### 3.4 Genetic diversity

The number of alleles per locus varied from 3 to 11, and null alleles were estimated to be <0.16% across all loci. Each of the eleven loci tested were found to be in gametic equilibrium. Tests of linkage disequilibrium, within and across populations, were all insignificant, satisfying the assumption that all loci are unlinked. The measures of diversity (allelic richness and gene diversity) decreased longitudinally, with the lowest levels of observed diversity in the westernmost populations. Heterozygosity, gene diversity, and allelic richness all followed this east – west pattern (Table 6). *F*_is_ scores for each of the clusters loosely followed this pattern and ranged from 0.18 to 0.06. Pairwise *F*_st_ scores ranged from 0.05 to 0.30, and all were significant (Table 7). The Mantel test showed Pearson's *r* of .68 that was highly significant (*p* = .001) meaning that individuals are more likely to find mates from populations geographically close to themselves rather than at random across all populations.

**Table 6:** Number of individuals (*N*), mean allelic richness (*A*_r_), gene diversity (*H*_s_), observed heterozygosity (*H*_o_), expected heterozygosity (*H*_e_), and the inbreeding coefficient (*F*_is_) for *Arctosa sanctaerosae* in five population clusters along the Northern Gulf of Mexico Coast

Population Cluster	*N*	*A*_r_	±95% C.I.	*H*_s_	±95% C.I.	*H*_o_	*SD*	*H*_e_	*SD*	*F*_is_
1	92	1.84	0.73	0.14	0.14	0.13	0.21	0.14	0.21	0.06
2	46	2.09	0.86	0.23	0.16	0.21	0.21	0.23	0.23	0.07
3	48	2.11	0.60	0.28	0.17	0.24	0.23	0.27	0.26	0.11
4	27	2.73	0.80	0.37	0.14	0.33	0.18	0.36	0.21	0.09
5	60	3.56	1.26	0.54	0.11	0.43	0.18	0.53	0.20	0.18

**Table 7:** Pairwise *F*_st_ Scores below diagonal and pairwise geographic distances (km) above the line

Population cluster	1.	2.	3.	4.	5.
1.	*	40.56	87.93	149.79	237.07
2.	0.0773	*	25.40	87.62	178.71
3.	0.1459	0.0464	*	23.18	119.35
4.	0.1577	0.0421	0.0179	^*^	98.08
5.	0.2891	0.1696	0.1250	0.0530	^*^

^*^ are blank values.

### 3.5 Historic and recent geneflow estimation

Using MIGRATE, a mean historic migration rate was calculated as the number of effective migrants (Table 8). BAYESASS estimates of recent migration were significantly lower, suggesting these clusters showed higher levels of isolation in the recent past. A paired *t* test of the means found the difference between historic and recent migration rates of *A. sanctaerosae* to be statistically significant (*df* = 4, *p* < .002).

**Table 8:** Comparison of the mean number of effective immigrants per generation reaching a population recently (as estimated by BAYESASS) versus the mean number of immigrants reaching a site historically (as estimated by MIGRATE)

Population cluster	Nm recent	Nm historic
1	0.01	1.23
2	0.57	2.68
3	0.14	2.75
4	0.69	3.34
5	0.09	1.28

### 3.6 Historic and recent bottleneck detection

Tests of heterozygosity using the statistical package BOTTLENECK showed no significant excess in the five clusters. However, the M ratios (generated by ARLEQUIN) ranged from 0.17 to 0.72. The three western clusters all had upper 95% C.I. estimates below the critical value of 0.68. These results support recent and severe bottleneck events in clusters one, two, and three. Cluster four has an M ratio below the critical value, but its confidence intervals overlap it suggesting an older or less severe event. Cluster five has an M ratio above the critical value and does not appear to have been bottlenecked.

### 3.7 Historic and recent effective population size estimation

Historic estimates of *N*_e_ (Table 5) across methods varied. The mean of the three methods was used for subsequent analyses and was significantly higher than the estimate of recent effective population size and the lower C.I. did not cross the upper C.I. of the estimates of recent *N*_e_.

Estimates of recent effective population size from LDNe varied widely and included negative values as well as upper estimates that reached infinity. Varying the lowest allele frequency from 0.01 to 0.02 did not narrow the 95% confidence intervals (Table 5). ONeSAMP gave results that appeared to be more biologically significant. Each of the clusters has a *N*_e_ of less than one hundred individuals with the exception of cluster 5. Mean estimates range from 32 to 153 individuals with less variation around the estimates using this method compared to LDNe. Only the ONeSAMP estimates were used for comparisons against historic *N*_e_.

## 4 DISCUSSION

The impact of hurricanes on the density of *A. sanctaerosae* was direct, density‐independent, and the best predictor of the magnitude of population reduction was the distance from the point of landfall. The effects of Hurricane Katrina showed a gradient of severity ranging from the most severe in the western sites to no measurable effect in sites beyond 283 km from the site of landfall. Hurricane Ivan, by contrast, was centered directly over the western sites and was of sufficient severity to affect every site.

More specifically, population clusters 2 and 4 are receiving more effective migrants than the other three clusters. The rate of emigration into Cluster 4 is best explained by its geographic isolation and small size in relation to the other populations, suggesting this locality may serve as a sink population that requires immigration to persist. The severity of effects experienced by Cluster 2 due to hurricanes in the recent past could explain the high number of migrants from Cluster 1. Additionally, the spatial heterogeneity of hurricane effects is not limited to the distance from landfall. The hurricane's leading edge has higher wind speeds relative to the trailing edge and is formed on the eastern side of the storm in the northern hemisphere. This frequently leads to an increased number of tornadoes spawned by the storm and more destructive power to the east of the storms point of landfall (Williams & Sheets, 2001). Hurricane Ivan made landfall due west of Cluster 2, and thus, the severity of effects may have been higher than that at Cluster 1, which with its high migration rate, provided a rescue effect. This migration appears to be elevated after major storm events.

The model that best explained population recovery in the year following a storm included both distance from landfall of the hurricane and the recovery of dune height. This dune habitat that is required for population recovery is often removed or highly partitioned by commercial development along the NGC. Five genetically distinct population clusters were recovered, and the barriers separating them were the high traffic beach communities of the NGC including the following: Gulf Shores, AL; Orange Beach, AL; Pensacola Beach, FL; Destin, FL; and Panama City Beach, FL; (Figure 1). This suggests that the commercial development of the NGC and the storm‐related population reductions work in concert to reduce the size of population clusters, reduce the cluster's ability to recover from storms, and reduce the population cluster's connectivity.

This type of habitat fragmentation shapes population genetic structure by reducing population cluster connectivity. In addition to low rates of gene flow, reductions in population size and loss of available high‐quality habitat can lead to genetic isolation and eventual extinction of populations (Reed 2008). Previous studies of spider population demography in fragmented habitats have found levels of structure and inbreeding similar to those found in the current study suggesting a decreased role of ballooning (Reed et al., 2011). The genetic isolation seen in the population clusters of *Arctosa sanctaerosae*is is likely due to a decline in effective population size and effective migration in the recent past with *F*_is_ scores ranging from 0.18 to 0.06, which suggests low amounts of dispersal across available habitat.

Historically, these population clusters experience severe reduction in size due to hurricanes or other catastrophic events; however, they had the ability to recover within one to two generations through migration to recently vacated habitat patches. This migration erased enough of the genetic signature of the reduction that it is undetectable using the current methodology. The genetic data suggest that there has been a significant decrease in the amount of migration between the five clusters within the last 100 years. However, this lack of migration in the recent past combined with the correlation of the patterns of genetic structure and the timing of the development of high traffic beach communities supports placing the timing of the subdivision to within the last 100 years. Once subdivided, the migration between clusters was reduced, and genetic drift, direct removal of diversity due to severe tropical storms, and commercial development led to declining population sizes and reduced diversity.

The observed genetic variation decreases along a geographic gradient from east to west with the alleles found in western populations as subsets of the alleles found in the eastern populations. Allelic richness and gene diversity are highest in the east and decrease as you move westward. The best three possible explanations for the higher genetic diversity seen in the eastern clusters are (a) the relatively few numbers of hurricanes to make landfall in the east in recent years, (b) significantly fewer high traffic beach communities, or (c) the possibility that *A. sanctaerosae* has expanded its distribution westward over time from the site of its divergence from its sister taxa. Hurricane Michael made landfall in October of 2018 in the eastern most cluster, which exhibits the highest richness and diversity, at Mexico Beach, Florida, and directly on several collection sites. The effects this will have on the species’ persistence would not be known for decades.

All of this data combined paints a picture of a metapopulation that has evolved in the presence of severe tropical storms and has been recently impacted through commercial development that has created barriers to repopulation, gene flow, and directly lowered *N*_e_. With the severity of tropical storms predicted to increase over time due to elevations in surface temperature of the Gulf of Mexico caused by climate change (Goldenberg et al., 2001; Slott et al., 2006; Webster et al., 2005), population connectivity will become increasingly important for population and even species persistence.

Moving forward, conservation measures must recognize the need for a contiguous dune system, not only for the physical preservation of the coastal dune ecosystem, but also for maintaining population structure and connectivity of this and presumably other species. Importantly, increased continuity and protection of these dunes will also lead to a healthier dune system that prevents erosion and inland destruction in the face of tropical storms. It must be recognized that a contiguous dune system, while beneficial to the focal species, also serves the broader goals of ecological and commercial interests along the NGC. By removing the dunes for commercial development, we have increased erosion and must reclaim the beach sands regularly as well as replace structures damaged during major tropical storms at great cost. It makes ecological as well as financial sense to restore these dune systems to their original state and allow natural processes to maintain them. Only the halting and/or reversal of the current developmental trends, including the complete removal of barriers interrupting the corridors for inter‐population migration, will result in the long‐term persistence of *A. sanctaerosae*.

## CONFLICT OF INTEREST

There are no sources or any potential sources of conflict of interest. There is no interest or relationship, financial or otherwise, that might be perceived as influencing the objectivity of this work.

## AUTHOR CONTRIBUTION

**Robert A Hataway:** Conceptualization (equal); Data curation (equal); Formal analysis (equal); Investigation (equal); Methodology (equal); Project administration (equal); Visualization (equal); Writing‐original draft (equal); Writing‐review & editing (equal). **David H Reed:** Conceptualization (equal); Formal analysis (equal); Methodology (equal); Supervision (equal); Writing‐original draft (equal).

## Data Availability

Microsatellite genotypes: https://doi.org/10.5061/dryad.m0cfxpp1z. Sampling locations and microsatellite genotypes available Dryad.
